# Principle Investigation and Method Standardization of Inhibition Zone Assay Based on Antimicrobial Peptides Extracted from Black Soldier Fly Larvae

**DOI:** 10.3390/biotech13030031

**Published:** 2024-08-09

**Authors:** Wenyue Shen, Ranxia Xue, Yanxia Liu, Shibo Sun, Xi Chen, Dongye Sun, Han Ouyang, Yuxin Li, Jianqiang Xu, Xiaoying Dong, Fengyun Ji, Weiping Xu

**Affiliations:** 1 School of Chemical Engineering, Ocean, and Life Sciences & Panjin Institute of Industrial Technology, Dalian University of Technology, Panjin Campus, Panjin 124221, China; shenwy99@mail.dlut.edu.cn (W.S.); jianqiang.xu@dlut.edu.cn (J.X.);; 2 Instrumental Analysis and Research Center, Dalian University of Technology, Panjin Campus, Panjin 124221, China

**Keywords:** black soldier fly, antimicrobial peptides, inhibition zone assay, diffusion, standardization, antimicrobial activity

## Abstract

The black soldier fly is a valuable resource insect capable of transforming organic waste while producing antimicrobial peptides (AMPs). The inhibition zone assay (IZA) is a method used to demonstrate the antimicrobial activity of AMPs. This study aimed to examine the experimental principles and establish a standardized IZA method. Results indicated that the AMPs extract consisted of proteins ranging in molecular weights from 0 to 40 kDa. The AMPs diffused radially on an agar plate through an Oxford cup. The diffusion radius was influenced by the concentration and volume of the AMPs but ultimately determined by the mass of the AMPs. The swabbing method was found to be effective for inoculating bacteria on the agar plate. Among the conditions tested, the plate nutrient concentration was the most sensitive factor for the IZA, followed by bacterial concentration and incubation time. Optimal conditions for the IZA included a nutrient plate of 0.5× TSA, a bacterial concentration of 10^6^ CFU/mL, and an incubation time of 12 h, with oxytetracycline (OTC) at 0.01 mg/mL serving as the positive control. The antimicrobial-specific activity of AMPs could be standardized by the ratio of inhibition zone diameters between AMPs and OTC. These findings contribute to the standardization of the IZA method for profiling the antimicrobial activity of AMPs.

## 1. Introduction

The black soldier fly (BSF, *Hermetia illucens*) is a resource insect that can convert animal manure and food waste into insect organic matter and fertilizer [1,2]. During this transformation process, the BSF can produce protein molecules with antimicrobial activity, known as antimicrobial peptides (AMPs) [3,4]. Genomic studies have revealed that the black soldier fly has the highest number and variety of AMP genes among known insects, including cecropins, defensins, attacins, lysozymes, etc. [3,4,5]. Due to the various properties of BSF AMPs, such as antibacterial, anti-inflammatory, and antibiofilm activities [3,6], they have attracted wide attention and investigation [7,8,9].

Antimicrobial activity is a characteristic feature of BSF AMPs. Common in vitro methods for detecting antimicrobial activity include the half inhibitory concentration (IC_50_) assay, minimum inhibitory concentration (MIC) assay, and inhibition zone assay (IZA) [10,11,12,13]. The IZA is often used as important evidence and a basis for comparing the antimicrobial activity of AMPs due to its intuitive nature. However, the standardization of IZA analysis has been limited by the technique’s difficulty and the lack of research on its experimental principles.

There are several challenges of IZA. This includes (1) the use of low electroosmotic or low-melting agarose gel in constructing the detection plate [14,15,16], which has created difficulties in bacterial inoculation [14,15,16]. The availability and cost of low electroosmotic or low-melting agarose gel are limiting factors, restricting the number and scale of experiments. Additionally, some microorganisms have a maximum temperature tolerance, making inoculation difficult when they do not tolerate the solidification temperature of the agarose gel; (2) there are substantial differences in the inoculation methods of bacteria and AMPs. For bacterial inoculation, methods such as using a triangular rake to spread the plate, mixing bacteria with agarose and pouring the plate, and using a cotton swab to streak the plate have been reported [14,17,18]. Methods such as the paper disk method of adding AMPs to thick filter papers and the punch method of adding AMPs to a hole in the plate have been reported [15,19]. Optimizing the inoculation methods is necessary for IZA analysis; (3) the optimal conditions for IZA are still unclear. There have been substantial differences in nutrient concentrations of agar plates, bacterial inoculation concentrations, and incubation times in IZA analysis [12,13,15], but there has been no systematic comparison of these factors nor exploration of the sensitivity and optimization range of these conditions; (4) the IZA lacks standardized methods, and experimental results may fluctuate randomly due to factors such as bacterial batch, culture time, and culture conditions, necessitating the introduction of a stable positive control to correct the variability of experimental results. Antibiotics approved for use in breeding animals could serve as a good positive control for the AMPs, as AMPs are considered potential candidates for antibiotic substitutes in animals [7,9].

The objective of this study is to standardize the IZA method and analyze its experimental principles. This study plans to profile the molecular weight distribution of AMPs through electrophoresis, demonstrate the diffusion property of AMPs in agar plates, compare the inoculation method of bacteria, optimize the experimental conditions of IZA, and finally establish a standardized method using antibiotic controls for calculating the specific antimicrobial activity of AMPs. Oxytetracycline (OTC), a member of the tetracycline antibiotics and permitted for use in breeding animals [7], is used as the antibiotic control.

## 2. Materials and Methods

### 2.1. Source of Larvae and Bacteria

Eggs of BSF were purchased from Langhao Environmental Technology Co., Ltd. (Nanjing, Jiangsu, China). Approximately 25 g BSF eggs were hatched in 500 g of wheat bran (70% moisture) at 27 °C for 7 days. Small larvae were separated by 2 mm sieves and used for food waste treatment. The strains of *Escherichia coli* ATCC25922 and *Bacillus subtilis* CCTCCM2021184 were retrieved from storage at −80 °C and cultured in Tryptone soy broth (TSB; Sangon Biotech Co., Ltd., Shanghai, China) at 30 °C for 16 h prior to use.

### 2.2. Larvae Rearing and Protein Extraction

The larvae were fed with food waste from the campus cafeteria. For every 450 g of food waste, 50 g of wheat bran was added. Solid NaOH was then added in the following varying amounts: 0 g, 0.16 g, 0.72 g, 2.35 g, and 7.74 g, respectively, and the mixture was stirred thoroughly. The pH value was measured in a 1:10 (*w*/*v*) deionized water dilution using a pH meter (FE38; Mettler-Toledo GmbH, Zurich, Switzerland). The initial pH values were found to be 3.95, 4.16, 6.59, 7.34, and 9.62, respectively. The BSF groups were therefore named BK (pH 3.95), pH 4.16, pH 6.59, pH 7.34, and pH 9.62. Each 500 g of the above food waste was placed in a plastic breeding box, and 1000 BSF larvae were added. The breeding boxes were kept at room temperature (23–32 °C) for 10 days with daily stirring. After 10 days, the BSF larvae were separated from the frass and allowed to defecate for one day before being stored at −20 °C.

Prior to AMP extraction, the BSF larvae were thawed to room temperature. The larvae were then mixed with an extraction solution containing 10% acetic acid and 0.01 mol/L Na2EDTA at a ratio of 1:10 (*w*/*v*) and ground using a colloid mill (JM-L50 colloid mill; Huawei Co., Ltd., Wenzhou, Zhejiang, China) to obtain a primary homogenate. The homogenate was centrifuged at 3500 rpm for 25 min (TD5A-WS centrifuge; Xiangyi Laboratory Instrument Co., Ltd., Changsha, Hunan, China) to obtain the supernatant. The supernatant was recovered and freeze-dried in a −50 °C freeze dryer (SCIENTZ-12N/C, Ningbo Xinzhi Freeze-drying Equipment Co., Ltd., Ningbo, Zhejiang, China). The resulting freeze-dried powder was the crude AMP extracts and was stored at −20 °C.

### 2.3. Electrophoresis and Staining

The AMP extracts were prepared as 0.10 g/mL solution using 0.10 g of freeze-dried powder mixed with 1 mL of deionized water. The mixture was centrifuged at 10,000 rpm for 3 min, and the supernatant was recovered as the AMP solution. For the sodium dodecyl sulfate polyacrylamide gel electrophoresis (SDS-PAGE), a Tricine SDS-PAGE gel was prepared according to Schägger et al. [20]. This involved preparing a 5 mL separating gel with 16.5% T and 3% C solution, a 0.8 mL stacking gel with 10% T and 3% C solution, and a 2 mL concentrating gel with 4% T and 3% C solution. These three gel solutions were sequentially added into an 8 cm × 8 cm × 1 mm glass gel plate to obtain the Tricine SDS-PAGE gel. Thereafter, 10 μL of the AMP solution was mixed with 5 μL of loading buffer [20] and incubated at 40 °C for 30 min prior to the loading. A GoldBand 3-color Low Range Protein Marker (2.7–40 kDa) (Yisheng Biotechnology Co., Ltd., Shanghai, China) was used as the molecular weight marker with 4 μL loaded. The electrophoresis was conducted using a JY-SCZ2+ mini electrophoresis tank (Junyi Electrophoresis Co., Ltd., Beijing, China) at 30 V for 1 h and 100 V for 6 h. After electrophoresis, the gel was fixed in a solution of 50% methanol and 10% acetic acid for 30 min, stained in a 0.05% Coomassie Brilliant Blue R250 and 10% acetic acid solution for 1 h, and then destained in a 10% acetic acid solution for 12 h before photography [20].

For the protein diffusion experiment, a nutrient-free plate was prepared using 1.5% agar powder. Three Oxford cups, stainless steel cylindrical rings with an outer diameter of 7.8 mm, inner diameter of 6.0 mm, and height of 10 mm, were placed on the surface of the plate. Then, 100 μL of the BK AMP solution was added to each cup. The maximum volume for an Oxford cup is 250 μL. The plate containing the Oxford cups was then incubated at 30 °C for 12 h. After incubation, the Oxford cups were carefully removed, and the agar plate was stained using the same procedure as the SDS-PAGE gel.

### 2.4. Comparison of Inoculation Method

The following three methods were compared for the bacteria inoculation procedure: spreading, pouring, and swabbing. The spreading method utilized a triangular rake to spread 100 μL of *E. coli* solution on the plate. The pouring method involved adding 1 mL of *E. coli* onto the plate, withdrawing all the solution after 1 min, and air-drying the plate for 0.5 h. The swabbing method used a cotton swab to streak along the plate. The cotton swab was dipped into the *E. coli* solution once, and the streaking was conducted every 120° around the plate. All the *E. coli* solutions used were 10^6^ CFU/mL, derived from two times 1:10 dilutions of the 10^8^ CFU/mL *E. coli* overnight culture using 1× PBS buffer (Phosphate Buffered Saline, pH 7.4). All plates contained 1.5% TSB and 1.5% agar. Since the 1× TSA plate consisted of 3% TSB and 1.5% agar, this plate was referred to as a 0.5× TSA plate. After the *E. coli* inoculation, triplicate Oxford cups were placed onto each plate, and 100 μL of 0.10 g/mL AMP solution from the BK group was added into each cup. The plates containing the Oxford cups were then incubated at 30 °C for 12 h to develop and observe inhibition zones. The entire procedure of the swabbing method was recorded in three separate videos and provided as Supplemental Materials.

### 2.5. Comparison of Antibiotics and Antimicrobial Peptides

Oxytetracycline of 99.9% purity was purchased from Aladdin Biochemical Technology Co., Ltd. (Shanghai, China) and dissolved in deionized water to prepare concentrations of 1.0 mg/mL, 0.1 mg/mL, 0.01 mg/mL, and 0.001 mg/mL. Solutions of AMPs were prepared from the BK sample using deionized water to achieve concentrations of 0.05 g/mL, 0.10 g/mL, 0.15 g/mL, and 0.20 g/mL.

For the IZA, streak lines were made on 0.5× TSA plates with 10^6^ CFU/mL of *E. coli* as described above, followed by the placement of four Oxford cups. Each cup received 100 μL of OTC solution at different concentrations, or 100 μL of AMP solution at different concentrations, or 50 μL, 100 μL, 150 μL, and 200 μL of 0.10 g/mL AMP solution. The plates containing Oxford cups were incubated at 30 °C for 12 h to develop and observe inhibition zones. Diameters of inhibition zones were measured using a vernier caliper. Each condition was conducted in triplicate.

For the diffusion assay, the plate was not inoculated with any bacteria or broth. A nutrient-free plate was prepared using 1.5% agar, and four Oxford cups were placed onto each plate. Each cup received 100 μL of OTC solution at different concentrations, or 100 μL of AMP solution at different concentrations, or 50 μL, 100 μL, 150 μL, and 200 μL of 0.10 g/mL AMP solution. The plates containing Oxford cups were incubated at 30 °C for 12 h. The plates containing OTC were imaged under ultraviolet light. The plates containing AMPs were stained as described for SDS-PAGE gels. Diameters of diffusion zones were measured using a vernier caliper. Each condition was conducted in triplicate.

### 2.6. Optimization of Inhibition Zone Assay

Optimization of IZA was conducted for nutrient concentration, *E. coli* concentration, and incubation time. A uniform 0.10 g/mL AMP solution from BK samples was prepared, and a uniform 0.01 g/mL OTC solution was used as a positive control.

For the nutrient gradient experiment, TSA medium was prepared at different concentrations, including 0.2× TSA (0.6% TSB, 1.5% agar), 0.5× TSA (1.5% TSB, 1.5% agar), 1× TSA (3% TSB, 1.5% agar), and 1.5× TSA (4.5% TSB, 1.5% agar). Each TSA plate was streaked with 10^6^ CFU/mL *E. coli* and loaded with triplicate Oxford cups. 100 μL of AMP or OTC solution was added into each cup, and the plates were incubated at 30 °C for 12 h before measuring the inhibition zones.

For the bacterial gradient experiment, *E. coli* solutions were diluted to 10^7^ CFU/mL, 10^6^ CFU/mL, and 10^5^ CFU/mL, respectively, from the overnight culture of 10^8^ CFU/mL with 1× PBS buffer. The 0.5× TSA (1.5% TSB, 1.5% agar) plate was streaked with each *E. coli* solution. Triplicate Oxford cups were loaded onto each plate, and 100 μL of AMP or OTC solution was added to each cup. The plates were incubated at 30 °C for 12 h before measuring the inhibition zones.

For the incubation time gradient experiment, plates of 0.5× TSA medium (1.5% TSB, 1.5% agar) were streaked with 10^6^ CFU/mL *E. coli* solution. Triplicate Oxford cups were placed on the plates with 100 μL of AMP or OTC added into each cup. The plates were incubated at 30 °C for 8 h, 12 h, 16 h, and 24 h, respectively. The diameters of the inhibition zones were observed and measured afterward.

### 2.7. Standardization and Application of Inhibition Zone Assay

The AMPs extracted from BSF reared in the BK (pH 3.95), pH 4.16, pH 6.59, pH 7.34, and pH 9.62 groups were used for standardization and application in the inhibition zone assay. Plates of 0.5× TSA medium (1.5% TSB, 1.5% agar) were streaked with 10^6^ CFU/mL *E. coli* or 10^6^ CFU/mL *B. subtilis* solutions, which were diluted from the 10^8^ CFU/mL overnight culture using 1× PBS buffer. Oxford cups were placed onto the plates and loaded with either 100 μL of 0.10 g/mL AMP solution or 0.01 mg/mL OTC solution. Triplicate tests were conducted for each AMP or OTC solution. The plates containing Oxford cups were incubated at 30 °C for 12 h, and the inhibition zone diameters were measured.

For the standardization of IZA, the diameter of the inhibition zone produced by 100 μL of 0.01 mg/mL OTC was defined as 1 U. The specific activity of each AMP against *E. coli* or *B. subtilis* was calculated using the following formula:

(1) $$Specific\;Activity\left(U/mg\right)=\frac{{Diameter}_{AMP}}{{Diameter}_{OTC}}\times 1U÷(V\times C)$$

where Diameter*_AMP_* and Diameter*_OTC_* are the inhibition zone diameters of the AMP and OTC, respectively (unit:mm); *V* is the volume of the AMP solution (unit:mL); and *C* is the concentration of the AMP solution (unit:mg/mL).

### 2.8. Statistical Analyses

Statistical analysis was conducted using SPSS Statistics version 22 (IBM Inc., Armonk, NY, USA) to compare the differences between the experimental groups with one-way analysis of variance (ANOVA). A difference with *p* < 0.05 was considered significant.

## 3. Results and Discussion

### 3.1. Electrophoresis and Diffusion of Proteins

**Figure 1:**
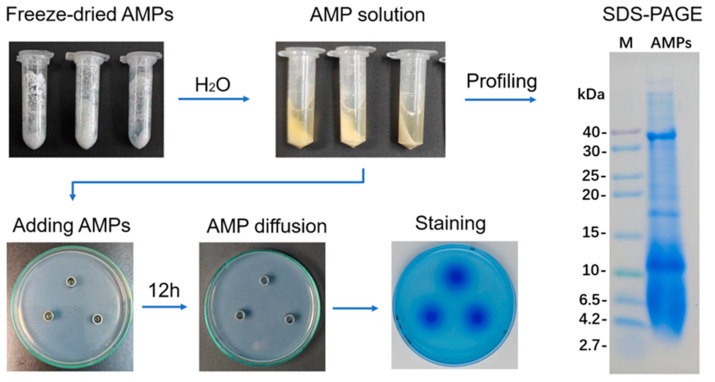
Preparation, electrophoresis, diffusion, and staining of antimicrobial peptides (AMPs).

The freeze-dried AMP extract appeared as a white powder (Figure 1). Upon reconstitution, some proteins dissolved, forming a light yellow solution, while others remained undissolved, resulting in a white precipitate after centrifugation (Figure 1). Tricine SDS-PAGE electrophoresis of the AMP solution revealed a protein molecular weight distribution mainly between 0–40 kDa, with few protein bands larger than 40 kDa, indicating that the reconstituted proteins were primarily small proteins with molecular weights less than 40 kDa. In the protein diffusion experiment, after overnight incubation for 12 h, the 100 μL of AMP solution in the Oxford cup was absorbed by the agar plate, leaving no liquid in the cup (Figure 1). After staining with Coomassie Brilliant Blue, the protein diffused on the agar plate showed a concentration gradient pattern. The protein inside the cup appeared to be a high concentration and uniformly deep blue, while the protein outside the cup showed a gradual decrease in concentration, transitioning from deep to light blue (Figure 1).

Previous studies have shown that acetic acid homogenate resulted in a protein solution with a continuous molecular weight range of 0–100 kDa [11], whereas reconstitution of freeze-dried acetic acid extracts resulted in protein molecular weights ranging from 0–40 kDa [10]. This study confirms these findings, showing that the re-dissolved AMP solution mainly consisted of 0–40 kDa proteins, suggesting that the undissolved precipitated proteins contain 40–100 kDa proteins. The AMPs of the BSF larvae mainly consist of small proteins, including defensins approximately 9–10 kDa, cecropins approximately 6–7 kDa, attacins approximately 20 kDa, and lysozymes approximately 16–17 kDa [3,6,10,11]. The current AMP solution includes these molecular weights of AMPs, but the components of the precipitated protein were not further investigated. Interestingly, Zdybicka-Barabas et al. [21] reported a similar phenomenon in which total proteins extracted from the hemolymph of BSF displayed a molecular distribution between 0–97.4 kDa, whereas AMPs extracted by methanol-acetic acid solution showed molecular weights between 0–30 kDa. Scieuzo et al. [18] also found that AMPs extracted by methanol-acetic acid had molecular weights between 0–15 kDa. These results suggest that the acetic acid extraction method may extract total proteins during the extraction process, but only small molecular weight proteins are re-dissolved during freeze-drying reconstitution, whereas the methanol-acetic acid extraction method may selectively extract small proteins during the extraction process. It can be confirmed that both the acetic acid [10,16] and methanol-acetic acid [18,21] extraction methods yield proteins with antibacterial activity, making them crude extracts of AMPs containing non-AMP proteins.

This study also utilized nutrient-free agar to demonstrate protein diffusion properties. Figure 1 illustrates that the protein concentration is high inside the Oxford cup, with a radial decrease outside the cup. It is clear that the formation of this gradient distribution is not immediate. After several hours of diffusion, the protein solution inside the Oxford cup is absorbed by the agar medium underneath or around the cup through capillary action, resulting in a radial concentration decay. In the subsequent IZA experiments, the growth of microorganisms and the diffusion of AMPs are synchronized over time. Therefore, it can be inferred that the phenomenon of inhibition zones outside the Oxford cup is influenced by multiple factors, such as the speed of protein diffusion, the diffusion concentration of AMPs, and the molecular composition of the AMP protein pool. Only when the AMPs reach an effective diffusion concentration within a limited diffusion time can they inhibit microbial growth and form inhibition zones.

### 3.2. Microbial Inoculation Methods

**Figure 2:**
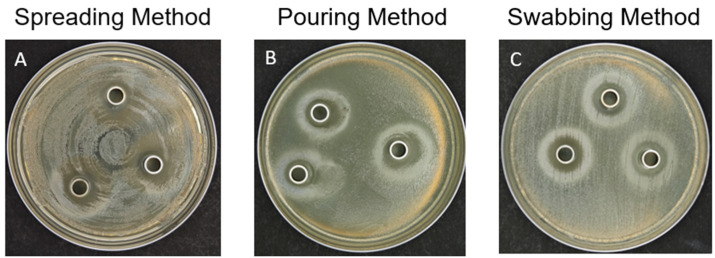
Comparison of bacterial inoculation method in the inhibition zone assay.

The inhibition zones of the AMPs obtained by the triangular spreader method show that the margins of the inhibition zones are not clear and do not form a distinct circle (Figure 2A). The inhibition zones obtained by the pouring method exhibit a locally irregular curve, with signs of bacteria being pushed aside at the periphery, making it difficult to measure the diameter of the inhibition zone (Figure 2B). In contrast, the cotton swab streaking method, i.e., the swabbing method, results in a clear, circular, transparent inhibition zone around the Oxford cup, with a white halo at the outer edge of the inhibition zone (Figure 2C). The halo may result from the competition between *Escherichia coli* and the AMPs. As shown in Figure 1, the AMPs form a diffusion zone of gradient concentration around the Oxford cup, while the white halo may result from an insufficient concentration of the AMPs, leaving traces of regrowth of *E. coli* after the digestion of AMPs.

As shown in Figure 2, the spreading method may not easily form a thin, translucent bacterial colony due to the uneven concentration of bacteria after spreading, resulting in unclear inhibition zones [17]. In the pouring method, this study used the method of pouring the bacterial liquid onto the plate surface and then collecting it back, while previous studies mainly used the method of adding the bacterial liquid to a low osmotic-pressure or low melting-point agarose and then pouring the bacteria-agarose onto the plate [13,14,15,22,23]. The latter methods are more likely to form clear, thin, translucent inhibition zones, being better than the current pouring method, but are limited by the following two conditions: (1) the tolerance temperature of the bacteria, as if the tolerance temperature of the test bacteria is lower than the solidification point of the low osmotic-pressure (35–37 °C) or low melting-point (26–30 °C) agarose, a large number of bacteria may die during plate preparation; (2) the need to purchase low osmotic-pressure or low melting-point agarose, which is more expensive than the agar powder used in this study, thus limiting the research scale. The advantage of the current swabbing method is that it produces the most clear and regular inhibition zones among the three inoculation methods, and it does not require low osmotic-pressure or low melting-point agarose, only using agar powder and requires practice in streaking technique. Therefore, the current study used the swabbing method in the following evaluations.

### 3.3. Evaluation of Antibiotics and Antimicrobial Peptides

**Figure 3:**
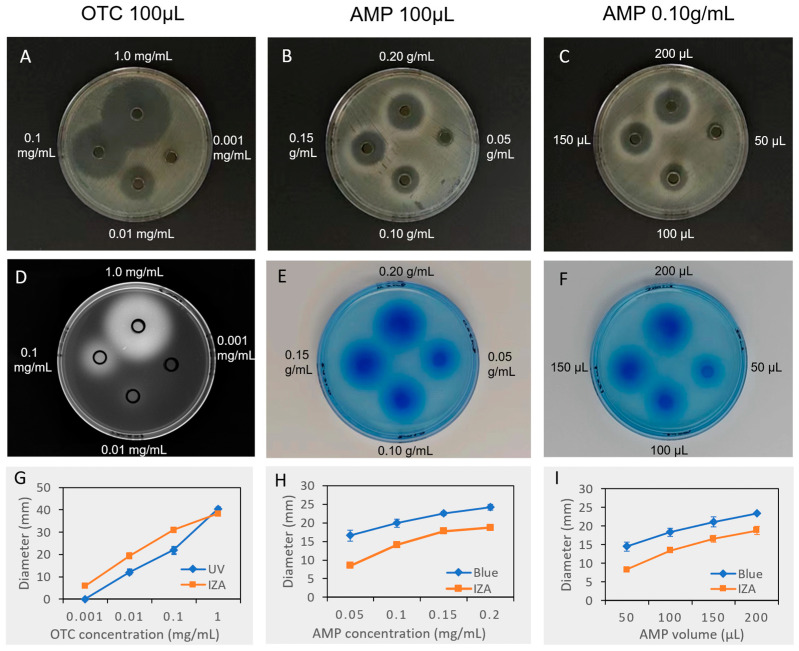
Comparison of inhibition and diffusion effect of antibiotics and antimicrobial peptides. (**A**–**C**) inhibition effect of oxytetracycline or antimicrobial peptides with concentration or volume gradient; (**D**) diffusion effect of oxytetracycline under UC-light; (**E**,**F**) diffusion effect of antimicrobial peptides stained by Coomassie brilliant blue; (**G**–**I**) measurement of diameters, *n* = 3. OTC, oxytetracycline; AMP, antimicrobial peptide; IZA, inhibition zone assay. Data are expressed as the mean of triplicate tests, with error bars representing the standard deviation.

The evaluation of the inhibition zone between oxytetracycline (OTC) and AMPs shows that OTC has a strong inhibitory effect against *E. coli*, with a low concentration of 0.01 mg/mL producing a significant inhibitory effect (Figure 3A), while the AMPs need to reach at least 0.10 g/mL of 100 μL to produce an inhibition zone comparable to 0.01 mg/mL OTC (Figure 3B,C). In the analysis of OTC, the inhibition zones (Figure 3A) were larger than the diffusion zones (Figure 3D) under the same concentrations (Figure 3G), indicating that the detection sensitivity of UV light is lower than that of the inhibition zone. In contrast, the inhibition zones of AMPs (Figure 3B,C) were smaller than the diffusion zones of Coomassie Brilliant Blue staining (Figure 3E,F), suggesting that the diffusion of AMPs resulted in a concentration gradient decay, and only when the AMPs reach an effective concentration in the diffusion zone can they exert an inhibitory effect (Figure 3H,I). These results also explain the formation principles of the white halo outside the AMP inhibition zone. The outer diameter of the white halo is almost the same as the outer diameter of the diffusion zone (Figure 3B,C,E,F), indicating that the white halo is likely the regrowth of *E. coli* after digesting the ineffective AMP extracts.

Comparing the concentration and volume gradient of AMPs, both the increase of AMP concentration and volume resulted in the increase of the inhibition zone, and the diameter of the AMP inhibition zone is related to the mass of AMPs in the Oxford cup. The similar mass of AMPs led to the same diameters of inhibition zones (Figure 3H,I). For example, the diameter of the inhibition zone of 100 μL of 0.20 g/mL AMPs is 23.97 ± 0.05 mm, while that of 200 μL of 0.10 g/mL AMPs is 23.96 ± 0.06 mm, and there is no difference between the two (*p* = 0.786). This result indicates that using 100 μL of 0.10 g/mL AMPs for IZA analysis is a suitable initial condition. If the inhibition zone is not formed under this condition, repeating the experiment with 200 μL of 0.10 g/mL AMPs or 100 μL of 0.20 g/mL AMPs will have a similar effect. Furthermore, 100 μL of 0.01 mg/mL OTC is an appropriate positive control for AMPs because its inhibitory effect is clear and stable, and the diameter of the inhibition zone is close to that of 100 μL of 0.10 g/mL AMPs.

### 3.4. Optimization of Inhibition Zone Assay

**Figure 4:**
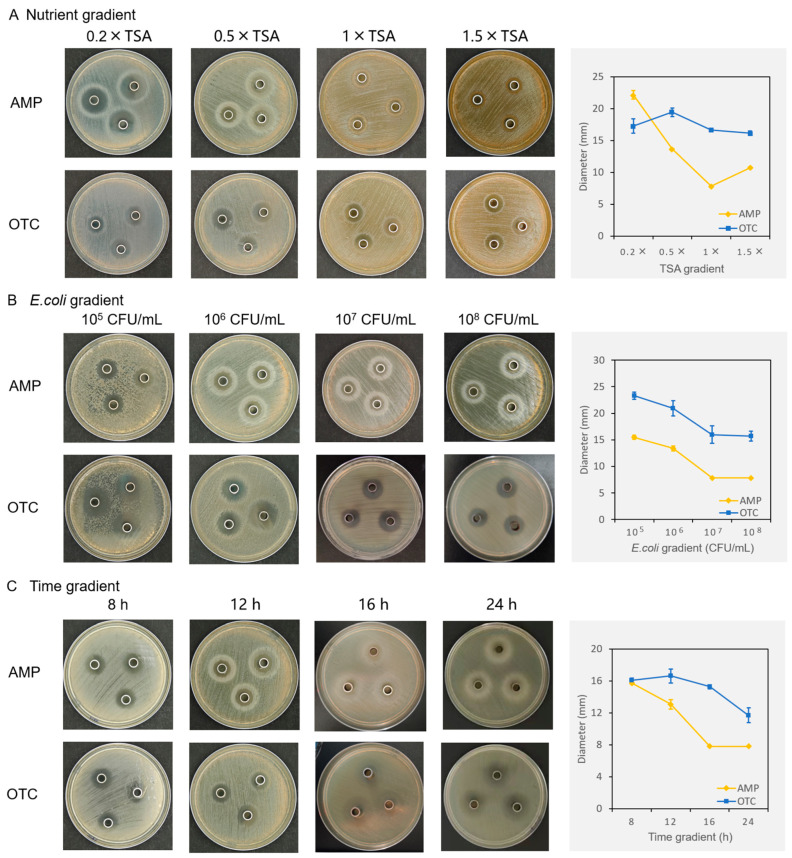
Method optimization of inhibition zone assay based on nutrient gradient (**A**), bacterial gradient (**B**), and incubation time gradient (**C**). AMP, antimicrobial peptide; OTC, oxytetracycline. Data are expressed as the mean of triplicate tests, with error bars representing the standard deviation.

The nutrient concentration, bacterial concentration, and culture time all significantly impact the diameters of the inhibition zones (Figure 4). Regarding nutrient concentration, the AMPs’ inhibition zone was largest on 0.2× TSA medium (*p* < 0.001), with a diameter of 22.15 mm. On 0.5× TSA medium, the diameter decreased to 13.67 mm, while on 1× TSA medium, the inhibition zone almost disappeared, with no *E. coli* growth in the Oxford cup, and the diameter was recorded as the outer diameter of the Oxford cup, 7.82 mm. On 1.5× TSA medium, the inhibition zone diameter was 10.76 mm (Figure 4A). Interestingly, the inhibition zones of OTC were less affected by the nutrient concentration than those of AMPs (Figure 4A), with diameters ranging from 16.17 to 19.44 mm, and the 0.5× TSA medium showed higher diameters than those of the rest medium (*p* = 0.029).

In terms of bacterial concentration, both the AMPs and OTC showed a trend of decreasing inhibition zones with increasing *E. coli* concentration (*p* < 0.05). As the *E. coli* concentration increased from 10^5^ CFU/mL to 10^8^ CFU/mL, the inhibition zone diameters of the AMPs decreased from 15.48 mm to 7.82 mm, and those of OTC decreased from 23.29 mm to 15.69 mm (Figure 4B).

In terms of culture time, both the AMPs and OTC exhibited a phenomenon of decreasing inhibition zones with prolonged culture time. For the AMPs, the inhibition zones were 15.73 mm at 8 h and decreased to 13.07 mm at 12 h, with a white halo appearing outside the inhibition zone. At 16 h and 24 h, the inhibition zone began to blur from the outside inward and became difficult to measure, so the diameters were recorded as the outer diameter of the Oxford cup, 7.82 mm (Figure 4C). For OTC, the inhibition zone also showed a phenomenon of shrinking and blurring from the outside inward at 16–24 h, with diameters of 15.27 mm at 16 h and 11.71 mm at 24 h (Figure 4C).

The inhibition zones of the AMPs decreased with increasing nutrient concentration, whereas OTC did not show a similar phenomenon (Figure 4A). These results indicate that AMPs and OTC have different mechanisms of microbial inhibitory effect. Antimicrobial peptides inhibit bacteria by disrupting cell membranes, as previously reported [7,8,9]. However, under moderate nutrient conditions, bacteria can produce proteases to digest AMPs, leading to the degradation of AMPs and the re-growth of bacteria. Oxytetracycline belongs to the tetracycline class of antibiotics, which inhibits the 30S subunit of the ribosome, occupies the A site for the aminoacyl tRNA in the ribosome, and inhibits bacterial protein translation [24,25]. The inhibitory effect of OTC is not affected by the substrate nutrient concentration, and *E. coli* is less likely to develop resistance to OTC with increased nutrient supply. Therefore, the inhibition zones of AMPs are significantly affected by the substrate nutrient level, while those of OTC are not.

Comparing the gradients of nutrients, bacteria, and time (Figure 4), the nutrient gradient produced the largest difference in the inhibition zones, indicating that it is the most sensitive condition for the IZA analysis of AMPs. However, only limited studies have used adjusting substrate nutrients for IZA analysis. Lee et al. [16] reported the use of low-nutrient medium (0.03% TSB, equivalent to 0.1× TSA in this study) mixed with bacterial solution to construct the lower agar plate and high-nutrient medium (6% TSB, equivalent to 2× TSA in this study) to construct the upper agar plate, resulting in clear inhibition zones.

Evidently, comparing the diameters of inhibition zones without limiting the medium nutrient concentrations cannot accurately reflect the inhibitory ability of the AMPs since the medium nutrient concentration is a critical condition for IZA analysis. Although 0.2× TSA medium can produce a more pronounced inhibition zone than 0.5× TSA medium, in order to be closer to the application scenario of AMPs, i.e., the scenario where AMPs are released in the digestive tract of feeding animals, this study selected 0.5× TSA medium as the standard medium for AMPs’ IZA analysis.

In the comparison of bacterial concentration and culture time, both the AMPs and OTC exhibited a phenomenon of inhibition zone shrinking or disappearing with increasing *E. coli* concentration and prolonged culture time (Figure 4B,C). This indicates that both the AMPs and OTC are at the lower limit of concentration, which can generate an inhibitory effect. When the bacterial concentration is increased or the culture time is prolonged, the bacteria on the periphery of the inhibition zone may spread inward, and the inactive bacteria within the inhibition zone may re-grow, resulting in the shrinking or blurring of the inhibition zone. For the AMPs, higher bacterial concentration and longer culture time increase the ability of bacteria to secrete proteases to hydrolyze the AMPs. For OTC, higher bacterial concentration reduces the amount of antibiotic allocated to individual bacteria, while longer culture time increases the ability of *E. coli* to develop resistance to OTC. Many previous studies used low-concentration bacteria for IZA analysis. For example, Park et al. [14,15] used a medium with 6 × 10^4^ CFU/mL bacteria to construct agar plates and detect inhibition zones, Choi et al. [19] used 40 μL of 2 × 10^6^ CFU/mL bacterial solution, and Choi et al. [12] used 30 μL of 5 × 10^6^ CFU/mL bacterial solution to conduct IZA analysis. This study also considers 10^6^ CFU/mL to be an appropriate bacterial concentration, which is more stable and reliable than 10^5^ CFU/mL for generating clear inhibitory zones.

Based on the results of nutrient, bacterial concentration, and time gradient comparisons, this study found that 0.5× TSA medium, 10^6^ CFU/mL bacterial solution, and 12 h culture time are appropriate conditions for IZA assays. Although those are not the condition with the largest inhibition zones, they are sensitive and suitable for forming clear and stable inhibition zones. Using OTC as a control helps to correct errors in different batch experiments and facilitates horizontal comparisons between different experimental platforms and different black soldier fly samples.

### 3.5. Standardization and Application of Inhibition Zone Assay

**Figure 5:**
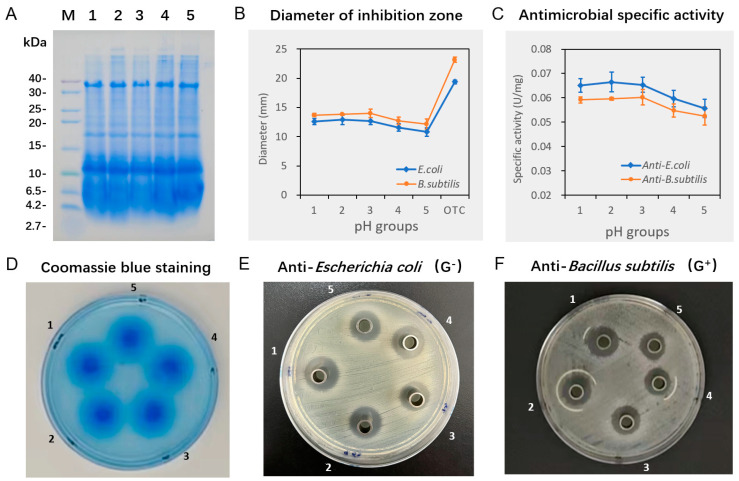
Characterization of antimicrobial peptides extracted from black soldier fly larvae reared in food waste with different initial pH. (**A**) tricine SDS-PAGE analysis; (**B**) diameters of inhibition zone; (**C**) specific activity of antimicrobial peptides against *E. coli* or *B. subtilis*; (**D**) diffusion zones of antimicrobial peptides; (**E**) inhibition zone assay against *E. coli*; (**F**) inhibition zone assay against *B. subtilis*. 1, BK (pH 3.95); 2, pH 4.16; 3, pH 6.59; 4, pH 7.34; and 5, pH 9.62 group. OTC, oxytetracycline. Data are expressed as the mean of triplicate tests, with error bars representing the standard deviation.

The results of AMP detection from black soldier flies at different pH values are depicted in Figure 5. The AMPs exhibited a molecular distribution ranging from 0–40 kDa (Figure 5A) and formed a diffusion circle with a diameter between 18.76–20.11 mm, showing a concentration radiating decrease on the diffusion plate (Figure 5D). In the anti-*E. coli* IZA detection, the inhibition zone diameter of the BK, pH 4.36, and pH 6.59 groups was 12.61–12.88 mm, which was slightly higher than the 10.78–11.56 mm of the pH 7.34 and pH 9.62 groups (*p* = 0.043, Figure 5B,E). The diameter of the OTC control group was 19.37 mm. For the anti-*B. subtilis* IZA detection, the inhibition zone diameter of the BK, pH 4.36, and pH 6.59 groups was 13.71–13.97 mm, slightly higher than the 12.18–12.72 mm of the pH 7.34 and pH 9.62 groups (*p* = 0.076, Figure 5B,F), with the diameter of the OTC control group being 23.18 mm. The specific activity against *E. coli* and *B. subtilis* exhibited similar trends as the diameters of the inhibition zones (Figure 5C), with detailed data illustrated in Table 1.

Based on the optimal conditions identified in Section 2.4, IZA detection was applied to the AMPs derived from BSF reared in different pH conditions. Both *E. coli* (G^−^) and *B. subtilis* (G^+^) assays showed clear and stable inhibition zones, and the specific activity of AMPs was evaluated by comparing them to the OTC control. The quantified results in Table 1 suggest that the initial pH of 3.95–6.59 has no significant impact on the antimicrobial activity of AMPs from BSF, but the initial pH of 7.34–9.62 resulted in a reduction of antimicrobial activity. Therefore, adjusting the initial pH value of food waste to an alkaline range with NaOH is not conducive to the activity of AMPs. This phenomenon may be attributed to changes in the nutrient content of food waste due to the increased NaOH content, or it may also derived from the changes in the microbial community generated from the addition of NaOH. Previously, Ma et al. [26] reported the results of breeding BSF at initial pH values of 2.0–10.0 and found that pH 2.0 significantly reduced the weight of BSF, while there was no weight difference among BSF at pH 4.0–10.0. The current results indicate that in the range of pH 4.0–10.0, an initial pH value of 3.95–6.59 was more favorable than 7.34–9.62 for the antimicrobial activity of BSF’s AMPs. Jin et al. [10] and Zhang et al. [11] reported the use of urea to adjust the C:N ratio of food waste and found that ratios of 18:1 and 16:1 were more beneficial than ratios of 14:1, 12:1, and 10:1 in the expression and activity of AMPs. This study aligns with those findings, indicating that only adjusting the initial pH or C:N of food waste to a moderate condition (pH 4–7, C:N 18:1–16:1) can greatly improve the activity and expression of AMPs.

## 4. Conclusions

This study elucidated the experimental principles of the inhibition zone assay through multiple approaches, including protein electrophoresis, diffusion tests, condition optimization, and method standardization. It established an optimized experimental method for the inhibition zone assay and a standardized method for calculating the specific activity of antimicrobial peptides based on antibiotic controls. The nutrient concentration of the plate was identified as the most sensitive condition for detecting the inhibition zone, followed by the bacterial concentration and the incubation time. The streaking method with a cotton swab was superior for bacterial inoculation. The conditions of 0.5× TSA medium, 10^6^ CFU/mL bacterial solution, and 12 h of incubation time were found to be suitable for detecting the inhibition zone, with 0.01 mg/mL oxytetracycline serving as a positive control. Based on the oxytetracycline control, the specific activity of antimicrobial peptides against different bacteria can be calculated. These findings are valuable for promoting the standardization of the inhibition zone assay and facilitating the comparison of microbial inhibition activity among different antimicrobial peptides and experimental platforms.

## Figures and Tables

**Table 1:** Inhibition zone diameters and specific activities of antimicrobial peptides extracted from black soldier fly larvae reared in food waste with different initial pH.

Groups ^1^	Anti-*E. coli* ^2^				Anti-*B. subtilis* ^2^			
	Inhibition Zone Diameter (mm)		Specific Activity (U/mg)		Inhibition Zone Diameter (mm)		Specific Activity (U/mg)	
	Mean	SD	Mean	SD	Mean	SD	Mean	SD
BK (pH 3.95)	12.61 ^ab^	0.53	0.0651 ^a^	0.0027	13.71 ^a^	0.29	0.0591 ^a^	0.0013
pH 4.16	12.88 ^a^	0.78	0.0665 ^a^	0.0040	13.82 ^a^	0.12	0.0596 ^a^	0.0005
pH 6.59	12.65 ^ab^	0.60	0.0653 ^a^	0.0031	13.97 ^a^	0.72	0.0603 ^a^	0.0031
pH 7.34	11.56 ^ab^	0.64	0.0597 ^a^	0.0033	12.72 ^a^	0.62	0.0549 ^a^	0.0027
pH 9.62	10.78 ^b^	0.72	0.0557 ^a^	0.0037	12.18 ^a^	0.88	0.0526 ^a^	0.0038
OTC	19.37	0.29			23.18	0.45		

^1^ Groups are divided by different initial pHs. OTC, oxytetracycline. ^2^ Values with different uppercase letters are significantly different; SD, standard deviation, *n* = 3.

## Data Availability

Data are available upon request to the corresponding author.

## Supplementary Materials

The following supporting information can be downloaded at https://www.mdpi.com/article/10.3390/biotech13030031/s1, Video S1: Streaking the plate with a cotton swab; Video S2: Loading the Oxford cup; Video S3: Adding the antimicrobial peptide solution.
